# Gender Based Salary Differences Among United States Academic Otolaryngologists

**DOI:** 10.1002/ohn.70202

**Published:** 2026-03-22

**Authors:** Larry W. Wang, Taryn E. Lubbers, Kirsten B. Burdett, Janice L. Farlow, Shirley Y. Su, Jessica H. Maxwell, Vanessa C. Stubbs, Katelyn O. Stepan

**Affiliations:** ^1^ Department of Otolaryngology‐Head and Neck Surgery Northwestern University Feinberg School of Medicine Chicago 60611 Illinois USA; ^2^ Department of Otolaryngology‐Head and Neck Surgery Texas Tech University Health Sciences Center School of Medicine Lubbock 79430 Texas USA; ^3^ Department of Preventative Medicine, Division of Biostatistics & Informatics Northwestern University Feinberg School of Medicine Chicago 60611 Illinois USA; ^4^ Department of Otolaryngology–Head and Neck Surgery Indiana University School of Medicine Indianapolis 46202 Indiana USA; ^5^ Department Head and Neck Surgery The University of Texas MD Anderson Cancer Center Houston 77030 Texas USA; ^6^ Department of Otolaryngology–Head and Neck Surgery University of Pittsburgh School of Medicine Pittsburgh 15213 Pennsylvania USA; ^7^ Department of Otolaryngology–Head and Neck Surgery Rush Medical College Chicago 60612 Illinois USA; ^8^ Department of Otolaryngology–Head and Neck Surgery Northwestern University Feinberg School of Medicine Chicago 60611 Illinois USA

**Keywords:** academic otolaryngology, gender difference, pay, salary

## Abstract

**Objective:**

To evaluate differences in financial compensation between male and female academic otolaryngologists.

**Study Design:**

Retrospective, cross‐sectional analysis.

**Setting:**

Multi‐institutional United States public medical schools.

**Methods:**

Twelve states with public salary data were identified, and otolaryngology salary data were extracted from associated academic institutions. Physician characteristics were extracted using Doximity, program website review, and direct faculty correspondence. Individual Medicare billing information was collected to account for differences in clinical effort. Univariable and multivariable linear regression models were performed to evaluate associations between gender and inflation‐adjusted salary before and after adjusting for physician characteristics, as well as Medicare billing after adjusting for research effort and administrative titles.

**Results:**

Data for 315 academic otolaryngologists was collected with 111 (35.2%) females and 204 males (64.8%). The median salary for females was $334,334 and $398,218 for males (*P* < .05). In the univariable linear regression setting, there was a significant difference in salary based on gender, with male surgeons making $90,000 more (*P* < .001). Following multivariable linear regression, faculty rank, number of publications as first or last author, and Medicare payment were all significantly associated with salary (*P* < .05). Male gender, number of publications as first or last author, and being a medical or residency/fellowship director were all associated with increased total Medicare payments in the multivariable linear regression setting.

**Conclusion:**

A significant difference in salary based on gender was observed for academic otolaryngologists in univariate analysis but not in multivariate analyses. However, total Medicare payment was significantly higher among men after controlling for physician characteristics.

Women are applying to medical school at higher rates than ever, comprising the majority of medical school applicants between 2019 and 2024.^1^ Despite the increase in women entering the workforce, studies show that gender disparities persist throughout academic medicine.^2^, ^3^, ^4^, ^5^ Many specialties, particularly surgical ones, have significant differences in compensation and leadership roles between genders even after accounting for age, faculty rank, research, and experience.^2^, ^3^ Women only hold a fraction of leadership positions in surgical subspecialties, making up 41% of faculty while only accounting for 25% of full professorships.^6^, ^7^, ^8^, ^9^, ^10^, ^11^

Academic otolaryngology is no exception to these trends. According to the Accreditation Council for Graduate Medical Education (ACGME), women in otolaryngology are less likely to hold leadership positions such as chair or program director.^12^ Gender discrepancies among leadership are especially pronounced in rhinology, laryngology, head and neck surgery, and facial plastics fellowship programs.^10^ While female otolaryngologists have the same number of career publications and citations as men in the position of fellowship director, they remain underrepresented in this area.^10^

Another important aspect of characterizing the gender gap within academic otolaryngology is financial compensation, which is becoming increasingly important as the number of female otolaryngologists rises.^10^ As such, this study seeks to evaluate differences in financial compensation between genders in academic otolaryngology.

## Methods

### Salary Data and Physician Characteristics

This study was exempt from the Northwestern Institutional Review Board.

Several states (California, Florida, Illinois, Kansas, Maryland, Michigan, North Carolina, Ohio, Tennessee, Texas, Washington, and Wisconsin) mandate the public release of state employee data including salary. We extracted salary data for academic otolaryngologists at 23 public medical schools in 12 geographically diverse states (University of Kansas; University of Wisconsin; University of Tennessee; Texas Tech University; MD Anderson Cancer Center; University of Texas Southwestern, San Antonio, Rio Grande Valley, Medical Branch, and Houston; University of Maryland; University of North Carolina; University of Washington; University of California, Davis, Los Angeles, San Diego, and San Francisco; University of Florida; University of South Florida; Ohio State University; Southern Illinois University; University of Illinois; University of Michigan) from govsalaries.com. Reported salary data ranged from 2022 to 2023 and all data were adjusted to 2023. Institutions with the majority of salary data below $100,000 were excluded (University of Kansas, University of Wisconsin, University of Tennessee, and University of South Florida).

Individual salary was combined with physician characteristics obtained from institutional websites and the online networking service, Doximity. Doximity aggregates information about US physicians from sources including the National Plan and Provider Enumeration System National Provider Identifier (NPI) Registry and self‐registered members without active NPIs. The use of Doximity has been previously validated, including a recent study assessing sex differences in academic contributions and networks among US academic otolaryngologists.^2^, ^4^, ^13^ Salary data was matched iteratively in accordance with methods previously described using the individual's first and last name, middle initial, and university affiliation.^2^, ^14^ Following matching, physician characteristics were obtained from Doximity: subspecialty, total Medicare payments in 2022 (cms.gov), faculty position, administrative title, number of years as an attending, number of PubMed publications, number of PubMed publications as first or last author, number of years since medical school, number of registered clinical trials (ClinicalTrials.gov), and number of NIH grants (NIH RePort database). Gender was identified using pronouns listed in faculty profiles. Faculty photo and name were used to identify gender when pronouns were not specifically stated. Medicare payments were utilized as an indicator for clinical productivity.

Administrative titles were identified through review of program websites and direct correspondence with department chairs or faculty. Individuals with executive leadership roles outside of Otolaryngology were excluded from analysis. Roles were further stratified into 3 categories: (1) medical directors, residency directors, associate residency directors, fellowship directors, associate fellowship directors, (2) vice chairs, and (3) division chiefs. This stratification was used to explore the impact of such roles on revenue generation. All physician characteristics including administrative titles, clinical trial involvement, NIH grants, and research output were obtained by September 2024.

### Statistical Analysis

Descriptive statistics were used to summarize physician characteristics. Continuous variables were reported using median, interquartile range (IQR), minimum, and maximum, and compared between groups using the Wilcoxon rank sum test. For categorical variables (clinical trials and NIH funding), frequencies and percentages were reported and compared using Fisher's exact test. Missing data were tabulated but not included in the descriptive analyses.

The association between salary and gender was assessed using linear regression models before and after adjusting for variables of interest. Multivariable models were adjusted for years since medical school, faculty rank, number of clinical trials, number of NIH grants, number of publications as first or last author, Medicare payments, and subspecialty as additive effects. Individuals with missing data were excluded from analyses. Subset analyses were performed to assess gender‐based pay differences by faculty rank. Similar analyses were performed to assess associations between total Medicare payments and gender within 3 groups related to administrative title ([1] residency/fellowship/medical directors or associate directors, [2] vice chairs, [3] division chiefs) and research effort (number of clinical trials involved in and NIH grants received). Prior to analyses, salary was scaled by $50,000 for interpretability, and salaries greater than $1 million or less than $100,000 were collapsed.

Several sensitivity analyses were also conducted. Analyses were conducted after excluding California and Texas given the disproportionate number of medical schools in these states. Additional sensitivity analyses were conducted with extreme salaries removed (>$1 million or <$100,000. All analyses were conducted at 0.05 significance level with unadjusted p‐values and corresponding 95% confidence intervals. Statistical analyses were performed using R v.4.2.2 statistical software (R Foundation).

## Results

### Characteristics of Study Population

315 academic otolaryngologists from 19 US medical schools were included in this study. 111 were women (35.2%) and 204 were men (64.8%) (Table 1). On univariable analysis, median salary for women ($334,334) was found to be $63,884 lower than that of men ($398,218) (*P* = .005). Female otolaryngologists also had significantly fewer years since completing medical school (median years, 16.0 women, 23.0 men, *P* < .001). While the distribution of faculty rank was not statistically significant, a higher percentage of women were assistant professors (40.5% women, 25.5% men) while more men were full professors (27.0% women, 41.2% men). There was no significant difference in number of NIH grants, though females had fewer publications (median, 23 vs 48.5, *P* < .001), publications as first/last author (median, 11 vs 21, *P* < .001), and having at least one registered clinical trial (median, 14 vs 51, *P* = .007).

**Table 1:** Physician Characteristics

Characteristic	Female, N = 111^a^	Male, N = 204^a^	*P*‐value^b^	Overall, N = 315^a^
Inflation‐adjusted salary in $			.005	
Median (IQR)	334,334 (200,000, 448,217)	398,218 (240,000, 609,203)		364,254 (222,517, 548,969)
Range	29,914, 1,000,000	39,347, 1,000,000		29,914, 1,000,000
Categorized inflation‐adjusted salary in $				
<100,000	8 (7.2%)	8 (3.9%)		16 (5.1%)
100,000‐199,999	19 (17.1%)	22 (10.8%)		41 (13.0%)
200,000‐299,999	18 (16.2%)	34 (16.7%)		52 (16.5%)
300,000‐399,999	29 (26.1%)	38 (18.6%)		67 (21.3%)
400,000‐499,999	15 (13.5%)	30 (14.7%)		45 (14.3%)
500,000‐599,999	10 (9.0%)	20 (9.8%)		30 (9.5%)
600,000‐699,999	3 (2.7%)	10 (4.9%)		13 (4.1%)
700,000‐799,999	0 (0.0%)	18 (8.8%)		18 (5.7%)
800,000‐899,999	2 (1.8%)	7 (3.4%)		9 (2.9%)
900,000‐999,999	5 (4.5%)	6 (2.9%)		11 (3.5%)
1,000,000+	2 (1.8%)	11 (5.4%)		13 (4.1%)
Medicare payment in $			<.001	
Median (IQR)	33,494 (1,671, 60,436)	57,494 (18,854, 97,643)		45,044 (10,130, 85,734)
Range	0, 191,595	0, 450,057		0, 450,057
Faculty rank			.010	
Assistant Professor	45 (40.5%)	52 (25.5%)		97 (30.8%)
Associate Professor	36 (32.4%)	68 (33.3%)		104 (33.0%)
Professor	30 (27.0%)	84 (41.2%)		114 (36.2%)
Years Since Medical School			<.001	
Median (IQR)	16 (12, 23)	23 (15, 32)		20 (14, 30)
Range	6, 44	7, 62		6, 62
Categorized years since medical school				
<10	14 (13.0%)	12 (5.9%)		26 (8.4%)
10‐14	38 (35.2%)	33 (16.3%)		71 (22.8%)
15‐19	18 (16.7%)	38 (18.7%)		56 (18.0%)
20‐24	13 (12.0%)	31 (15.3%)		44 (14.1%)
25‐29	9 (8.3%)	24 (11.8%)		33 (10.6%)
30‐34	10 (9.3%)	26 (12.8%)		36 (11.6%)
35+	6 (5.6%)	39 (19.2%)		45 (14.5%)
No. of publications			<.001	
Median (IQR)	23 (9.0, 47.0)	48.5 (19.5, 97.0)		36 (15.5, 80.0)
Range	0, 224	0, 425		0, 425
No. of publications as first or last author			<.001	
Median (IQR)	11 (4.0, 21.0)	21 (8.5, 39.0)		16 (6.3, 33.0)
Range	0, 102	0, 139		0, 139
Any registered clinical trials			.013	
No	95 (87.2%)	153 (75.0%)		248 (79.2%)
Yes	14 (12.8%)	51 (25.0%)		65 (20.8%)
No. of registered clinical trials			.007	
Median (IQR)	0 (0.0, 0.0)	0 (0.0, 0.3)		0 (0.0, 0.0)
Range	0, 11	0, 19		0, 19
Any NIH grants			.43	
No	95 (85.6%)	167 (81.9%)		262 (83.2%)
Yes	16 (14.4%)	37 (18.1%)		53 (16.8%)
No. of NIH grants			.42	
Median (IQR)	0.0 (0.0, 0.0)	0.0 (0.0, 0.0)		0.0 (0.0, 0.0)
Range	0, 21	0, 51		0, 51
Subspecialty			.021	
Facial Plastic and Reconstructive Surgery	10 (9.0%)	21 (10.3%)		31 (9.9%)
General	23 (20.7%)	24 (11.8%)		47 (15.0%)
Head and Neck	17 (15.3%)	53 (26.1%)		70 (22.3%)
Laryngology	12 (10.8%)	21 (10.3%)		33 (10.5%)
Otology & Neurotology	9 (8.1%)	29 (14.3%)		38 (12.1%)
Pediatric	28 (25.2%)	29 (14.3%)		57 (18.2%)
Rhinology and Skull Base Surgery	12 (10.8%)	26 (12.8%)		38 (12.1%)
Admin Titles			.51	
Division Head	11 (25.6%)	20 (24.1%)		31 (24.6%)
RD/FD/MD	22 (51.2%)	36 (43.4%)		58 (46.0%)
RD/FD/MD, Division Head	5 (11.6%)	7 (8.4%)		12 (9.5%)
RD/FD/MD, Vice Chair	1 (2.3%)	5 (6.0%)		6 (4.8%)
RD/FD/MD, Vice Chair, Division Head	1 (2.3%)	5 (6.0%)		6 (4.8%)
Vice Chair	3 (7.0%)	4 (4.8%)		7 (5.6%)
Vice Chair, Division Head	0 (0.0%)	6 (7.2%)		6 (4.8%)

Abbreviations: FD, fellowship director or associate director; MD, medical director or associate director; RD, residency director or associate director.

^a^ n (%).

^b^ Wilcoxon rank sum test; Fisher's exact test.

**Figure 1:**
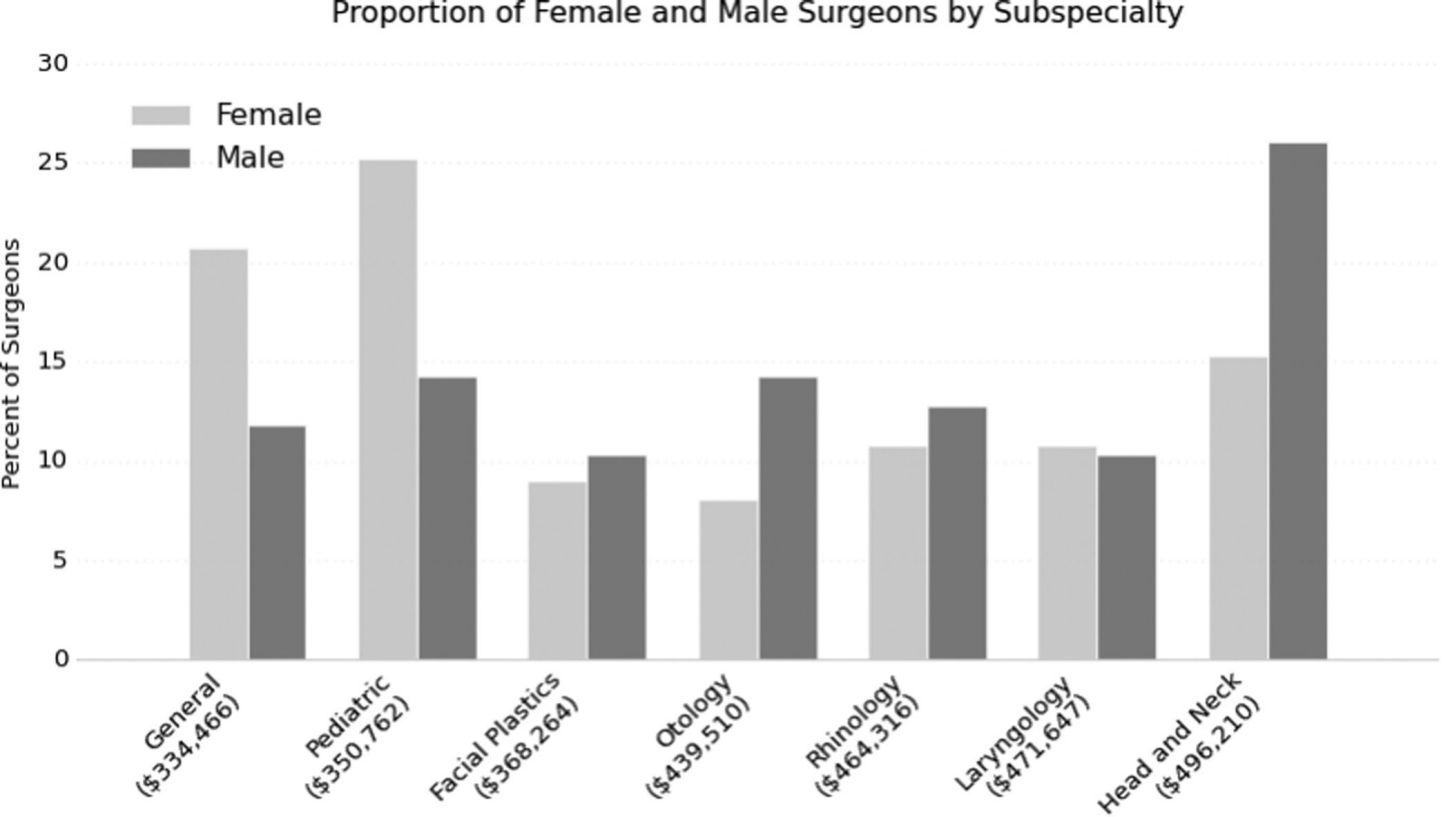
Proportion of male and female otolaryngology subspecialties and mean salary.

**Figure 2:**
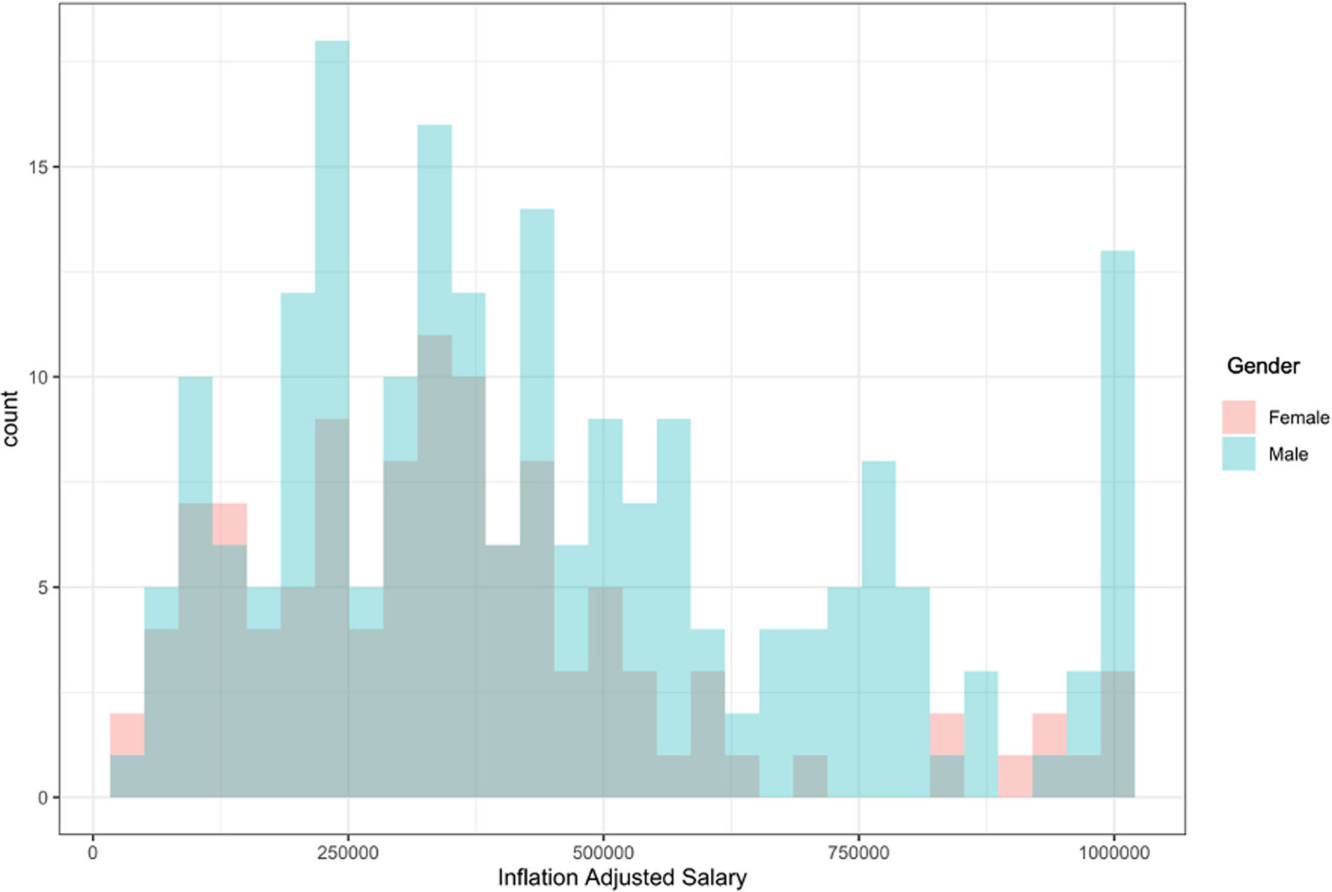
Academic otolaryngology salary by gender.

Mean annual salary was the lowest for general and pediatric otolaryngology ($334,467 and $350,763) and highest for head and neck surgery ($496,210). Subspecialty distribution between gender was significantly different (*P* < .05), with most women subspecializing in pediatric otolaryngology (25.2%) and most men in head and neck surgery (26.1%) (Figure 1). The salary distributions for men and women were similar across all subspecialties (Figure 2). However, most making more than $750,000 a year were men.

### Univariate and Multivariate Analyses

Following univariable analysis, annual salaries between men and women remained significantly different (Table 2). After adjusting for years since medical school, faculty rank, clinical trials, NIH funding, publications as first/last author, Medicare payments, and subspecialty, there was no significant difference in annual salaries between genders. Faculty rank, number of first/last author publications, and total Medicare payments were instead associated with increased salary for both genders. The mean adjusted salary difference between full and assistant professors was $120,000 (*P* = .002). For each first or last author publication, salary increased by $2000 (*P* = .001). Lastly, for each additional $50,000 in Medicare payments, salary increased by $75,000 (*P* < .001). After adjusting for administrative titles, publications, and total Medicare payments remained significantly associated with increased salary. There was no significant difference in salary between genders in subset analyses by faculty rank.

**Table 2:** Univariable and Multivariable Salary Regression Models for All Subspecialties

Characteristic	Univariable				Multivariable		
	N	Beta	95% CI	*P*‐value	Beta	95% CI	*P*‐value
Gender				.003			.476
Female	111	—	—		—	—	
Male	204	1.8	0.62, 2.9		−0.39	−1.4, 0.68	
Years since medical school	311	0.15	0.09, 0.20	<.001	0.03	−0.03, 0.10	.281
Faculty rank				<.001			.002
Assistant professor	97	—	—		—	—	
Associate professor	104	1.3	0.00, 2.6		0.25	−1.0, 1.5	
Professor	114	4.9	3.7, 6.2		2.4	0.85, 4.0	
No. of registered clinical trials	313	0.63	0.28, 0.98	<.001	0.16	−0.14, 0.47	.288
No. of NIH grants	315	0.23	0.12, 0.35	<.001	0.09	−0.02, 0.19	.098
No. of publications as first or last author	302	0.09	0.07, 0.10	<.001	0.04	0.01, 0.06	.001
Medicare payment in $ scaled by 50 K	315	1.7	1.3, 2.2	<.001	1.5	0.99, 1.9	<.001
Subspecialty				.002			.127
Facial plastic and reconstructive surgery	31	—	—		—	—	
General	47	−0.68	−2.9, 1.6		−0.34	−2.3, 1.6	
Head and neck	70	2.6	0.47, 4.7		1.7	−0.17, 3.5	
Laryngology	33	2.1	−0.36, 4.5		−0.35	−2.5, 1.8	
Otology & neurotology	38	1.4	−0.92, 3.8		−0.10	−2.1, 1.9	
Pediatric	57	−0.35	−2.5, 1.8		0.80	−1.1, 2.7	
Rhinology and skull base surgery	38	1.9	−0.43, 4.3		0.75	−1.3, 2.8	
No. Obs.					300		
AIC					1707		

Abbreviation: CI, confidence interval.

Across all administrative titles, total Medicare payments were significantly higher among men when controlling for years since medical school, clinical trial involvement, NIH funding, and first/last author publications (Table 3). On average, the mean adjusted total Medicare payment was $25,000 higher for men among all 3 groups. While there was no significant association between holding vice chair or department chair positions and increased Medicare payment, being residency/fellowship/medical director or associate director was associated with increased Medicare payment. The mean adjusted total Medicare payment was $30,500 higher among otolaryngologists holding any of these positions (*P* < .05).

**Table 3:** Association of Administrative/Educational Leadership Roles and Medicare Payments (Scaled by $50,000): Univariable and Multivariable Linear Regression

Characteristic	Univariable				Multivariable		
	N	Beta	95% CI1	*P*‐value	Beta	95% CI1	*P*‐value
Gender				<.001			<.001
Female	111	—	—		—	—	
Male	204	0.57	0.29, 0.84		0.51	0.22, 0.79	
Years since medical school	311	0.00	−0.01, 0.02	.7	‐0.01	−0.03, 0.00	.060
No. of registered clinical trials	313	0.06	−0.03, 0.14	.2	0.00	−0.08, 0.09	.915
No. of NIH grants	315	0.01	−0.01, 0.04	.3	0.00	−0.02, 0.03	.734
No. of publications as first or last author	302	0.01	0.01, 0.02	<.001	0.01	0.00, 0.02	<.001
Subspecialty				<.001			
Facial plastic and reconstructive surgery	31	—	—				
General	47	−0.21	−0.68, 0.26				
Head and neck	70	0.25	−0.19, 0.70				
Laryngology	33	1.0	0.51, 1.5				
Otology & Neurotology	38	0.01	−0.48, 0.51				
Pediatric	57	−1.2	−1.6, −0.72				
Rhinology and skull base surgery	38	0.40	−0.10, 0.89				
Residency, fellowship and medical director or associate director (collapsed)				<.001			<0.001
No	233	—	—		—	—	
Yes	82	0.68	0.39, 0.98		0.61	0.32, 0.91	
No. Obs.					301		
AIC					934		

Abbreviation: CI, confidence interval.

In sensitivity analyses, there was no significant gender difference in annual salary after excluding Texas and California schools or removing outlier salaries.

## Discussion

Utilizing data from US medical schools with public salaries and professional information from online databases, this study assessed gender differences in financial compensation among academic otolaryngologists. Although females were shown to have significantly lower salaries on univariate analysis, no significant difference was found on multivariate analysis. Faculty rank, publications, and Medicare payments were instead significantly associated with higher salaries, suggesting that the observed univariate difference may be attributable to differences in workforce composition. No difference was found in administrative titles between men and women. These findings may reflect a positive shift within academic otolaryngology, as compensation appears increasingly aligned with objective academic and clinical productivity metrics. These patterns may signal progress towards narrowing disparities as early‐career women advance into senior positions.

Some of our findings contrast with previous data on gender inequalities in academic settings. Prior studies have found a significant difference in pay between men and women even after accounting for the same variables considered in our study.^2^, ^3^ Jena et al found that among all specialties, female physicians earned $19,878 less than males across institution, faculty rank, and specialty.^2^ Many studies also found that women otolaryngologists were underrepresented in leadership positions and less likely to receive financial support for research.^10^, ^12^, ^15^, ^16^, ^17^, ^18^ In a recent study utilizing AAMC Faculty Salary Survey data for otolaryngologists, Shah et al found that women had a significantly lower median compensation across all faculty ranks besides Instructors.^19^ While both our study and the recent AAMC Faculty Salary Survey analysis identify gender‐based salary differences in academic otolaryngology, there are notable differences in methodology and scope. The AAMC analysis utilizes aggregate, self‐reported institutional data from a large national cohort and stratifies compensation trends by rank, gender, race/ethnicity, and geographic region. However, it does not account for individual‐level research productivity, clinical activity, or administrative roles, limiting its ability to explore potential explanatory factors for observed disparities.^19^ While our study is limited by its smaller sample and inability to capture non‐Medicare clinical work or nonsalary compensation, it uniquely integrates measures of both research and clinical activity, providing additional insight into salary inequities in academic otolaryngology.

One potential contributing factor to these differences in findings is pay transparency. Several states require governmental agencies to report salaries for all employees, including physicians employed at public medical schools. This transparency may contribute to greater pay equity by increasing accountability and reducing gender‐based compensation disparities. The absence of an adjusted gender salary difference in our analysis may reflect this influence. With a growing trend toward salary disclosure with increasing requirements for listed salary ranges in job postings and on employment platforms, greater pay transparency may help extend these equity benefits into private healthcare settings as well. Institutions that openly publish or standardize compensation by rank and role may be better positioned to minimize inequities and reduce the impact of implicit bias during salary negotiations. However, because this study was limited to institutions with publicly available data, it may not fully capture inequities at private institutions where compensation structures remain less transparent and subject to fewer regulations. Future work should therefore include private medical schools, hospitals, and independent practices to better understand how transparency affects compensation patterns across otolaryngology.

Another major consideration is the distribution of administrative assignments. Although this study did not find a significant difference in leadership positions, men and women held administrative titles at similar rates despite women being significantly earlier in their careers on average. One hypothesis for gender pay disparities is that female academic physicians are more likely to hold positions with significant administrative and teaching responsibilities, such as residency/fellowship/medical director or associate director. Some of these responsibilities, including slowing down operative or clinic workflows to teach, may reduce billable clinical volume, as demonstrated by studies showing decreased work relative value units (wRVU) generated per hour when attendings operated with trainees.^20^ However, other roles may increase clinical exposure, such as oversight of resident clinics or running multiple operating rooms, thereby increasing billable encounters. This heterogeneity may at least partially explain why these roles were associated with higher Medicare payments in our analysis despite their added administrative burden. Additionally, given Medicare payments reflect service volume and coding intensity, not just clinical time efficiency, there are likely several mechanisms independent of administrative positions that also contribute to higher payments generated among these groups.^21^, ^22^

While it is difficult to accurately assess how men and women differ in the amount of time and effort they devote to teaching and administrative responsibilities, this study attempted to gain more insight by looking at Medicare billing by gender, controlling for research effort (grants/trials) and administrative titles. Medicare billing is being used in this context as a proxy for revenue from performing clinical services, with the hypothesis that females may have lower clinical productivity (ie Medicare billing) when accounting for research efforts and administrative roles. Among all 3 groups examined ([1] residency/fellowship/medical directors and associate directors, [2] vice chairs, and [3] division chiefs], multivariable analysis found that females had significantly lower mean total Medicare payments by $25,000.

A potential driver for these gender differences in Medicare billing is the amount of time spent on patient care during and between visits.^23^ A study by Roter et al found that female physicians spent, on average, 2 minutes (10%) longer during medical visits than males—allowing them to engage in significantly more patient‐centered dialogue.^24^ Outside of patient rooms, female physicians also spend more time in electronic health record (EHR) documentation. In a study of 318 physicians, Rotenstein et al found that after adjusting for specialty, age, patient volume, and percentage of orders, women spent 41 more minutes in EHR than men. This included 10 more minutes working during nonscheduled hours and 31 more minutes writing notes. The increased time spent in EHR may be partially due to increased messages received, with Rittenberg et al demonstrating that female physicians receive 52 additional patient messages and 10 additional staff messages per month compared to males.^25^ While these factors have been shown to be associated with improved patient outcomes, seeing less patients overall due to the increased amount of time spent on each individual patient may lead to decreased billing.^26^

Referral patterns may further contribute to billing disparities. Chami et al demonstrated that physicians tend to refer patients to specialists of the same gender—with a 10% higher likelihood of male physicians referring to male specialists.^27^ As a result, male specialists average 633 referrals annually while female specialists averaged 433.^27^ The mean revenue per referral was also lower for females ($316 vs $350), resulting in female specialists earning 4.7% less than their male counterparts even after adjusting for specialty, tenure, academic status, and practice rurality.^27^ As such, future efforts should subsequently characterize gender differences in clinical output in relation to time, referrals, and other administrative roles and their potential influence on salary inequity.

Furthermore, different career trajectories between genders may also play a part in pay discrepancies. Although our study found that women had a similar number of grants as men, a trend described by Roman et al, another study suggests that women in academic otolaryngology exhibit a different productivity curve than their male counterparts, as measured by the h‐index, producing less research early in their careers but surpassing men in research productivity later in their careers.^16^, ^28^ Since financial compensation is often associated with research output, this could further contribute to salary differences. Female otolaryngologists may have a slower productivity rise due to a lack of mentors and sponsors, more participation in teaching/mentorship, academic responsibilities less associated with traditional academic productivity metrics such as publications, and maternity leave.^13^, ^16^ They may also have more responsibilities as the primary caregiver of their children, with Lyu et al finding 57.2% and 43.5% of female physicians report having the sole responsibility of routine and back‐up/emergency child care plans, respectively.^29^

There were several limitations associated with this study. First, salary data were only assessed from a portion of public medical schools. While these schools represent a geographically diverse sample of academic otolaryngologists, due to the limited available data, the present study did not incorporate data from other public schools, private medical schools, or community practices which may have gender differences in salaries. Additionally, only public salary data and Medicare payments were included as measures for financial compensation. Some institutions with collections‐based systems, varying payer mixes, other sources of income such as incentive pay, bonuses, or dual appointments across university and hospital systems may not have been fully captured or clearly delineated in public salary data, potentially skewing observed salary trends. Additionally, while Medicare payments provide a useful proxy for clinical effort, they may not precisely reflect true clinical full‐time equivalent. Several data sources were also used to collect physician characteristics for the multivariable analyses. This process may have resulted in data errors or omissions; however, data validation steps were implemented to mitigate these issues, making it unlikely that any gender was disproportionally affected. Lastly, for faculty members without included gender pronouns, the authors were limited to inferring gender from faculty name and profile photo. We recognize that this approach may not accurately represent nonbinary and gender expansive individuals, highlighting an important area for future studies.

## Conclusion

Our analysis found that female academic otolaryngologists had lower median salaries and Medicare payments than their male counterparts in unadjusted analyses. However, salary differences were not significant after adjusting for faculty rank, experience, administrative titles, and clinical and academic productivity metrics. Medicare payment disparities persisted, with women holding more administrative and teaching roles relative to years in practice, potentially impacting clinical output. While advancements have been made in addressing gender‐based salary disparities in otolaryngology, further research is needed to understand and address the underlying factors influencing career advancement and clinical productivity gaps.

## Author Contributions

**Larry Wang**: design, data acquisition, statistical analysis, data interpretation, drafting, and revision; **Taryn Lubbers**: design, data acquisition, statistical analysis, data interpretation, drafting, and revision; **Kirsten Burdett**: design, statistical analysis, data interpretation, drafting, and revision; **Janice Farlow**: design, statistical analysis, data interpretation, drafting, and revision; **Shirley Su**: design, statistical analysis, data interpretation, drafting, and revision; **Jessica Maxwell**: design, statistical analysis, data interpretation, drafting, and revision; **Vanessa Stubbs**: design, statistical analysis, data interpretation, drafting, and revision; **Katelyn O. Stepan**: design, data acquisition, statistical analysis, data interpretation, drafting, revision, and supervision.

## Disclosures

This study was presented at the 2025 Combined Otolaryngology Spring Meetings–American Head & Neck Society, May 14‐May 18, 2025, New Orleans.

### Competing interests

None.

### Funding source

None.
